# Fate Mapping Identifies the Origin of SHF/AHF Progenitors in the Chick Primitive Streak

**DOI:** 10.1371/journal.pone.0051948

**Published:** 2012-12-13

**Authors:** Esther Camp, Susanne Dietrich, Andrea Münsterberg

**Affiliations:** 1 School of Biological Sciences, University of East Anglia, Norwich Research Park, Norwich, United Kingdom; 2 Institute of Biomedical and Biomolecular Science, University of Portsmouth, Portsmouth, United Kingdom; Brigham & Women’s Hospital - Harvard Medical School, United States of America

## Abstract

Heart development depends on the spatio-temporally regulated contribution of progenitor cells from the primary, secondary and anterior heart fields. Primary heart field (PHF) cells are first recruited to form a linear heart tube; later, they contribute to the inflow myocardium of the four-chambered heart. Subsequently cells from the secondary (SHF) and anterior heart fields (AHF) are added to the heart tube and contribute to both the inflow and outflow myocardium. In amniotes, progenitors of the linear heart tube have been mapped to the anterior-middle region of the early primitive streak. After ingression, these cells are located within bilateral heart fields in the lateral plate mesoderm. On the other hand SHF/AHF field progenitors are situated anterior to the linear heart tube, however, the origin and location of these progenitors prior to the development of the heart tube remains elusive. Thus, an unresolved question in the process of cardiac development is where SHF/AHF progenitors originate from during gastrulation and whether they come from a region in the primitive streak distinct from that which generates the PHF. To determine the origin and location of SHF/AHF progenitors we used vital dye injection and tissue grafting experiments to map the location and ingression site of outflow myocardium progenitors in early primitive streak stage chicken embryos. Cells giving rise to the AHF ingressed from a rostral region of the primitive streak, termed region ‘A’. During development these cells were located in the cranial paraxial mesoderm and in the pharyngeal mesoderm. Furthermore we identified region ‘B’, located posterior to ‘A’, which gave rise to progenitors that contributed to the primary heart tube and the outflow tract. Our studies identify two regions in the early primitive streak, one which generates cells of the AHF and a second from which cardiac progenitors of the PHF and SHF emerge.

## Introduction

The heart is the first organ that becomes functional in the developing vertebrate embryo and initially appears in the ventral midline as a linear endocardial tube, ensheathed by myocardial cells. This primary linear heart tube develops from the ventral midline fusion of bilateral heart fields located in the lateral plate mesoderm. These bilateral heart fields, also known as cardiogenic mesoderm, consist of cardiac progenitor cells (CPCs), which are among the first cells during gastrulation to ingress from the primitive streak in amniotes [1]. A number of studies have focused on identifying the embryonic origins of cardiogenic mesoderm in amniotes, particularly chicken and mouse embryos, which, similar to human, develop a four-chambered heart. However, the majority of these studies have involved fate mapping experiments in the chick due to their accessibility and ease of manipulation *in ovo* and *ex ovo* which makes them well suited to analyse early cardiac lineage and cell fate [2], [3], [4], [5].

Fate map analyses using fluorescent dyes have shown that CPCs are located in the anterior-middle region of the primitive streak in stage Hamburger and Hamilton (HH) 3 chicken embryos [6], [2], [5]. Between stage HH3 and HH4 these cells gastrulate through the anterior-middle part of the primitive streak as part of the mesoderm [2]. CPCs move out bilaterally, away from the streak in an anterior-lateral direction and by HH 5-6 are located in the lateral plate mesoderm [7], [8], [9], [10]. When the lateral plate mesoderm splits into the somatic and splanchnic mesoderm, CPCs are restricted to the splanchnic mesoderm [11] and constitute the cardiogenic mesoderm also described as bilateral primary heart fields [7]. At HH7-8 the bilateral heart fields are drawn towards the midline at the anterior intestinal portal creating a crescent shape. This occurs due to complex morphogenetic movements involving the endoderm [12] and the extracellular matrix [13] along with the response of progenitor cells to chemotactic signals [9], [10]. As the anterior intestinal portal continues to move caudally, the splanchnic mesoderm folds ventrally and medially and fuses at the midline by HH9, resulting in the formation of the primary heart tube by HH10.

Once the primary heart tube has formed it begins to loop and to elongate concomitant with myocardium growth [14]. Classical marking experiments suggest that important structures of the outflow tract, such as the atrioventricular canal and conotruncus, are added secondarily to the straight heart tube during looping [15], [16]. Thus, elongation and growth of the heart tube is not only due to the expansion of the tissue already in the tube but also the addition of CPCs from the surrounding mesoderm after HH10.

It has been shown in chicken embryos that cells which make up the linear heart tube are from the primary heart field (PHF), the population of CPCs within the bilateral cardiac mesoderm which fuses at HH9 [16], [17]. In contrast, the myocardium of the conotruncus, the outflow myocardium (outflow tract), which is added after the formation of the primary heart tube, is derived from as secondary lineage of CPCs that are from heart fields designated as the secondary heart field (SHF, [17]) and the anterior heart field (AHF, [18]). By stage HH12-13, the SHF is situated in the splanchnic mesoderm that underlies the caudal pharynx and provides myocardium to the outflow tract during looping [17], whilst the AHF consists of undifferentiated cephalic mesoderm, in pharyngeal mesenchyme surrounding the aortic sac and immediately anterior to the linear heart tube [18]. The location of the AHF is corroborated by the identification of a pool of undifferentiated cardiogenic cells within the head mesoderm in stage HH16 embryos [19]. In vertebrates, head muscles are derived from the cranial paraxial mesoderm (CPM, [20], [21]) and fate maps between stage HH8-22 have demonstrated that cells from the CPM that migrate to the first pharyngeal arch also contribute to the myocardial and endocardial layers of the outflow myocardium [19]. It is interesting to note that the SHF is thought to be a subdomain of the AHF [22]. However, the SHF is situated slightly caudal to the AHF and is restricted to the pre-pharyngal mesoderm, caudal to the outflow tract. The AHF covers a broader area and includes both the pre-pharyngeal mesenchyme and more anterior splanchnic mesoderm that extends into the middle of the cranial pharyngeal arches [23].

A second heart field has also been identified in mouse embryos [24]. This study reported a lacZ transgenic insertion into the mouse fibroblast growth factor 10 (Fgf10) locus, in which FGF10-lacZ expression was observed in the right ventricle, pharyngeal mesoderm and the outflow tract of the heart and in outflow tract progenitors. These progenitors were continuous with the splanchnic mesoderm and the mesodermal core of the pharyngeal arches [24]. These results suggest that in mice, the second heart field population of cardiac progenitor cells is situated in a similar location to that of the AHF in chicken embryos [18], [25].

Although the existence and location of the SHF and AHF in chicken embryos have been described previously, these observations have been made in embryos after the primary heart tube had already formed [17], [18]. A few studies have focused on identifying the location of CPCs from these heart fields before the formation of the linear heart tube. AHF progenitors have been mapped to the CPM in stage HH8 chicken embryos [19] a region that principally generates head skeletal muscle [20], [21]. In addition, cells that have been described to be AHF progenitors were found to be located in the dorsomedial region of the splanchnic mesoderm adjacent to the CPM in HH8 chicken embryos [25]. In this study AHF progenitors were observed to contribute to the distal part of the pharyngeal mesoderm as well as the cardiac outflow tract [25]. In addition, fate mapping in chicken embryos from HH8 onwards has shown that the cranial caudal polarity of the future heart tube is represented medio-laterally in the cardiac mesoderm of embryos [26]. Results from this study suggest that at stage HH8 the cardiogenic mesoderm is organised in craniocaudal stripes with the inflow myocardium precursors located in the most lateral part while the outflow myocardium precursors are located in the most medial part of cardiogenic mesoderm [26]. Thus, at stage HH8 CPCs of the SHF and AHF in the splanchnic mesoderm are contiguous with CPCs from the PHF and are adjacent to the CPM. Furthermore, the inflow myocardium of the heart is made up from PHF SHF and AHF progenitors. It appears that all CPCs are closely linked, yet the delineation of the different heart fields and the difference in timing of incorporation into the heart suggested the possibility that there might be an early segregation of the different CPCs lineages. However the origin, before HH8, of CPCs that contribute to the outflow myocardium has not been investigated.

Previously, a prospective fate mapping study of the avian cardiovascular system was performed on early primitive streak stage embryos using quail/chick transplantation chimeras and fluorescent dye injections [2]. Results from this study demonstrated that cells from the anterior-middle primitive streak contributed to the heart [2]. Furthermore, it has been observed that CPCs, which ingress from this region of the primitive streak, contribute to the myocardial and endocardial layers of the linear heart by stage HH9 [5]. This led us to consider whether a discrete population of CPCs, which will constitute the SHF/AHF, could be identified in early primitive streak stage chicken embryos.

We injected vital fluorescent dyes and performed transplants with GFP fluorescent grafts to analyse the potential ingression site of cardiogenic progenitor cells within the primitive streak. These experiments were performed on chicken stage HH3 through to HH3^+^ embryos in modified early chick (EC) culture [27] or in a modified cornish pasty culture (MC) [28]. Using these approaches we have generated a prospective fate map of cells, which were located within the CPM by stage HH8-10 and in the pharyngeal mesoderm by stage HH15, and of CPCs, which remained undifferentiated within the splanchnic mesoderm at stage HH12 and contributed the OFT by stage HH15. Our results demonstrate that progenitor cells which contribute to the CPM and pharyngeal mesoderm ingress during stage HH3 through to HH3^+^ through a rostral region of the anterior-middle primitive streak, defined as region ‘A’. Previous fate maps between stage HH8-22 have demonstrated that cells from the CPM that migrate to the first branchial arch are progenitors of the AHF and contribute to the outflow myocardium [19], [25]. Furthermore, our fate mapping studies reveal for the first time that in chicken embryos CPCs of the prospective PHF, SHF and part of the AHF ingress simultaneously from the caudal region of the anterior-middle primitive streak, defined as region ‘B’. We show that by HH12 region ‘B’ derived progenitors reside in the splanchnic mesoderm and the linear heart tube and by HH15 contribute to the outflow myocardium. We propose that during early stages of development this population of region ‘B’ derived progenitors is present within the bilateral cardiogenic mesoderm as a cohort. We found that upon convergence and fusion of the cardiac mesoderm at the midline of the embryo at HH9-10 some CPCs from this population had differentiated and entered the heart, forming the primary heart tube, whereas other region ‘B’ derived CPCs were still undifferentiated and located in the splanchnic mesoderm. These progenitors are now referred to as being from the SHF/AHF, which will differentiate and be added to the developing heart later in development.

## Materials and Methods

### Embryos and Nomenclature

Wild type fertilized chicken eggs (Winter Egg Farm, Royston, Hertfordshire. UK) and CAG-GFP fluorescent fertilized chicken eggs (The Roslin Institute, Edinburg. UK [29]) were incubated at 38°C in a humidified incubator for 12–14 hours. Embryos were removed from the yolk and prepared for EC culture [27] and staged according to Hamburger and Hamilton (HH) [30] or Primitive Streak stages (PS) [31]. Embryos were left to develop in EC culture or in MC culture. Embryos for MC culture were prepared as described [28].

### Primitive Streak Injections with Vital Fluorescent Dyes

Embryos ranging from HH3 (PS2) through to HH3^+^ (PS4) in EC were examined using Zeiss Stemi SVG dissecting microscope (with 0.63 objective and 5× magnification) and pressure injected with either 3,3-dilinoleyloxacarbocyanine perchlorate (DIO, Molecular Probes, D275), DiI (Molecular Probes, C-7001) or both. These fluorescent dyes are lipophilic and lable cell membranes and other hydrophobic structures. DiI at a final concentration of 1 mg/ml in ethanol was mixed with an equal volume of 0.3 M sucrose in PBS and warmed to 37°C before injection. DiO was injected at 2.5 mg/ml in DMF and warmed to 37°C before injection. Dyes were loaded into a glass capillary needle and injected into the primitive streak using an Eppendorf Femtojet microinjector. A Zeiss eye piece reticule with a NE1scale (100 subdivisions of 0.1 mm) was used to measure the position of the injection of vital dyes along the primitive streak. Position 0 of the scale was aligned to the most rostral end of the primitive streak and both the length of the entire primitive streak and the position of vital dye injection were recorded. Fluorescent dyes were injected in subdivisions of 31.7 µM in length within two regions of the anterior-middle primitive streak, a rostral region (‘A’) and caudal region (‘B’). Embryos were repeatedly washed post injection with PBS to ensure that excess dye was removed.

### Primitive Streak Homotopic and Isochronic Grafts

Small grafts of primitive streak were dissected from region A or region B of HH 3 (PS 2) though to HH 3^+^ (PS4) donor CAG-GFP fluorescent embryos and transferred to region A or region B of unlabelled, stage-matched host embryos. To determine correct position of region A and B, grafts were transferred using the same Zeiss eye piece reticule and measurement technique as for primitive streak vital fluorescent dye injections. After grafting, host embryos were incubated in a humid chamber for 30 minutes at 28°C to allow the embryo to heal and the grafted tissue to be incorporated.

### Histology, Time-lapse Imaging and Immunohistochemistry

Embryos were immediately examined after vital fluorescent dye injection or grafting under a fluorescence microscope (to confirm point of injection or location of graft) and then incubated in a humid chamber at 38°C until they reached the desired stage. During development, embryos were examined to determine the position of the labelled cells and their descendants. For time-lapse imaging, embryos were cultured in six-well dishes and mounted on an inverted wide-field microscope (Axiovert, Zeiss) and bright-field and fluorescent images were collected every 6 minutes over a period of 8 hours using Axiovision software. At the end of imaging, the data was exported as individual still frame TIFF files.

When embryos reached the desired stage they were fixed in 4%PFA/PBS and examined as whole mounts. Subsequently they were equilibrated in 30% sucrose in PBS and embedded in OCT (R1180, Agar Scientific) and prepared for cryo-sectioning. After sectioning, the position of vital fluorescent dye or GFP labelled cells was determined by microscopy. To detect Islet-1 (Isl1) protein, sections were blocked with 5% goat serum in 0.1% tween/PBS for 30 minutes. Primary antibody against Islet-1 (1∶200, 40.2D6, Developmental HybridomaBank) was used overnight at 4°C. After washing in 0.1% Tween/PBS, sections were incubated with anti-mouse-Alexa488-conjugated secondary antibody (1∶500, Molecular Probes). Sections were counterstained with 4′,6-diamidino-2-phenylindole (DAPI,32670, 1∶10,000, Sigma). Images were obtained with a Zeiss M2 Bio Quad SV11 fluorescence stereomicroscope, a Zeiss AxioplanZie upright microscope and confocal images with a Leica TCS SP2 UV system.

## Results

### Fate-mapping of Cells in HH3 to HH3^+^ Primitive Streak Embryos Defines New Regions ‘A’ and ‘B’

In HH 3 chicken embryos, cells within the anterior third of the primitive streak, but excluding Hensen’s node, contribute to the heart [2]. We set out to map the origin of CPCs within the primitive streak, which contribute to the outflow myocardium. We labelled primitive streak cells of HH3 to HH3^+^ embryos with the vital fluorescent dyes 3,3-dilinoleyloxacarbocyanine perchlorate (DIO) or DiI, which label the cell membrane. Sites within two distinct regions along the anterior-middle of the primitive streak were injected, a rostral region (region A) and caudal region (region B) (Table 1). Our defined regions ‘A’ and ‘B’ correlate with earlier mapping studies and partially overlap with regions of the primitive streak previously demonstrated to contribute to the heart [2]. However, the sites previously described were larger (125 µm) than our delineated regions, which were made up of subdivisions of 31.7 microns (µm) in length. The number of subdivisions within each region changed slightly during the stages examined, as during HH 3 through to HH 3^+^ the primitive streak increases in length.

**Table 1:** Limits of region A and B in early primitive streak embryos.

Stage of embryo	HH3* (PS2∧)	HH3* (PS3∧)	HH3^+^ * (PS4∧)
**Primitive streak length**	35 (1110 µm)	40 (1268 µm)	50 (1585 µm)
**Region A**	6–11 (n = 45)	6–11 (n = 54)	7–13 (n = 56)
**Region B**	12–15 (n = 64)	12–16 (n = 65)	14–17 (n = 64)

* Developmental stage indicated as Hamburger Hamilton (HH) stages [30].

∧ Developmental stage indicated as Primitive Streak (PS) stages [31].

Table indicates the length of the primitive streak during development from stage HH3 (PS2) to HH3^+^ (PS4) and the limits of region A and region B along the anterior-middle primitive streak. An eye piece reticule with 100 subdivisions (0 to100) was used to delineate region A and region B. The length of the primitive streak is shown in the number of subdivisions and in microns (µm). The limits of region A and region B for each developmental stage are shown as subdivisions. The primitive streak of all embryos used in the study was measured to determine developmental stage and a total of 348 embryos were labelled and analysed to determine the limits of each region. Labelling experiments were performed by either injecting vital fluorescent dyes at one to three subdivisions within each region or by grafting GFP fluorescent primitive streak tissue from most of region B of a donor embryo into the equivalent region B of a host wild type embryo.

**Figure 1:**
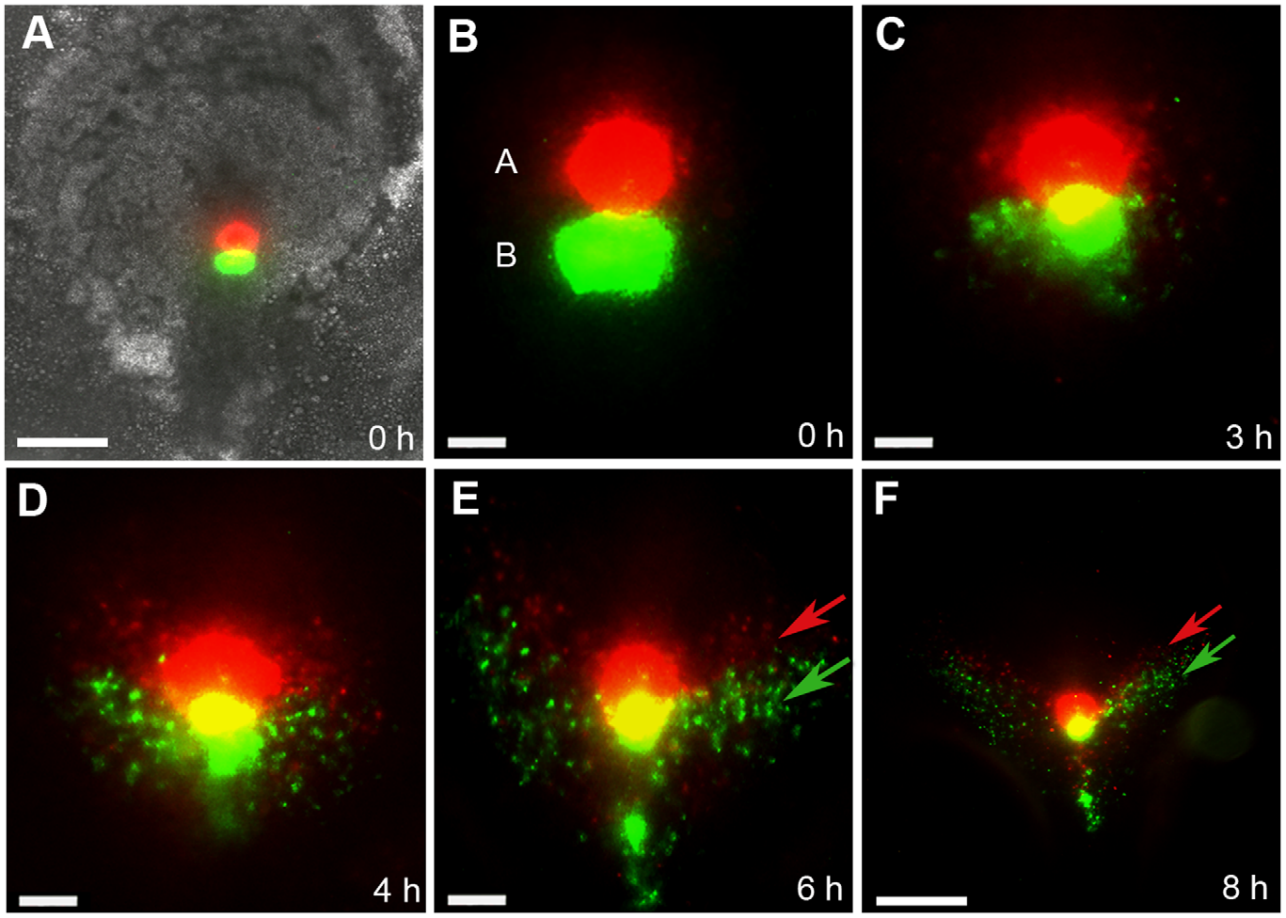
Ingression and early migration of cells from region A and B. (**A**) Bright field and fluorescent image of an early primitive streak stage embryo (HH3^+^, PS4) labelled with DiI (red) and DiO (green) along the anterior-middle primitive streak. (**B**) Close up image of fluorescently labelled cells within region A and B. (**B–F**) Time-lapse microscopy of the embryo in (**A**) revealed early movement patterns of labelled cells in region A and region B (n = 7). Panels represent still images obtained from time-lapse microscopy at the time points indicated in hours (h). (**C–F**) Cells from region A and B were found to migrate away from the primitive streak bilaterally in an anterior-lateral direction. Arrows in (**E–F**) indicate movement trajectories of cells from region A (red arrow) and region B (green arrow). Scale bars (**A**) and (**F**) = 100 µm, (**B–E**) = 50 µm.

**Figure 2:**
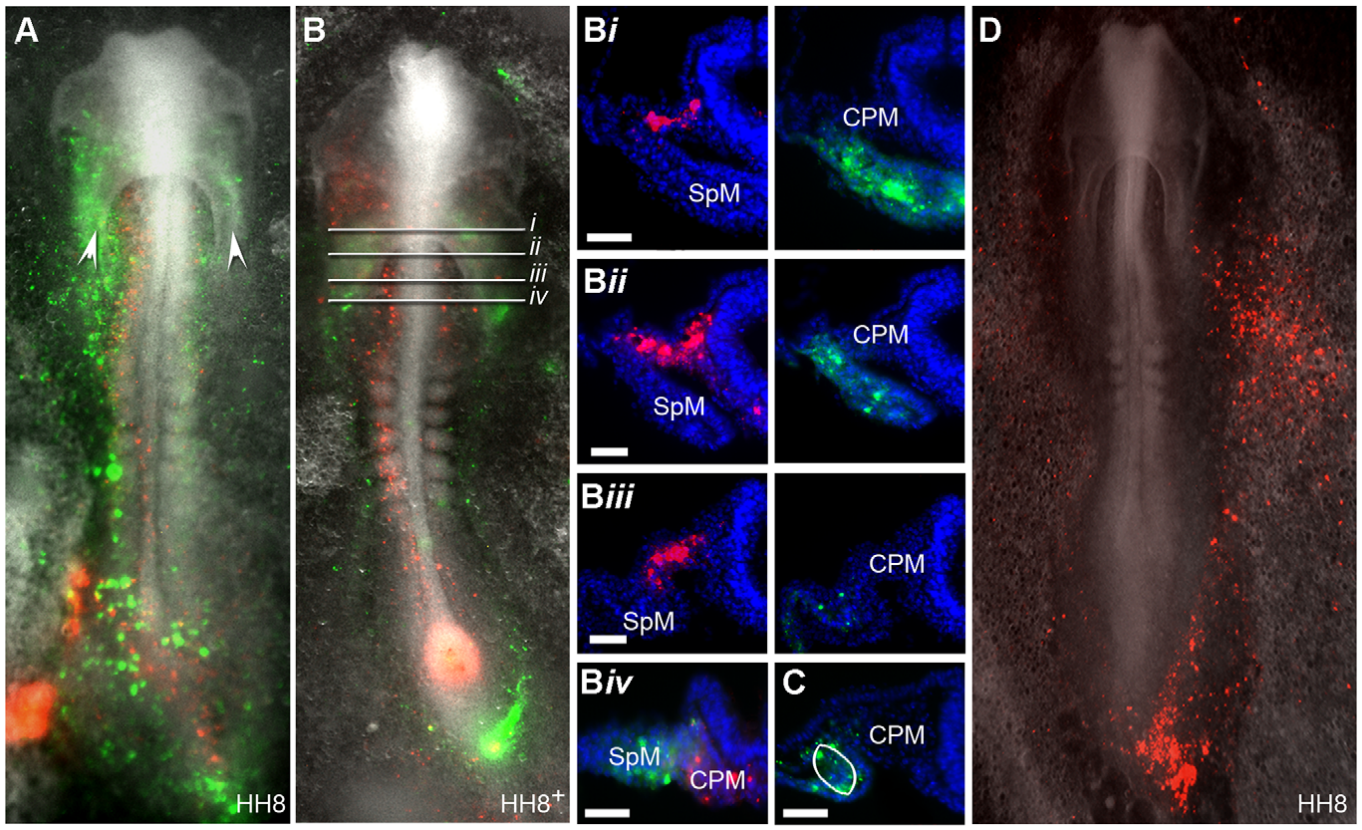
Location of fluorescent descendants of cells from region A and B at stage HH8. (**A–B**) Bright field and fluorescent images of labelled embryo at stage HH8 and HH8^+^ showing the location of fluorescently labelled cells. Descendants from region A are labelled with DiI (red) and descendants from region B are labelled with DiO (green). White arrow heads in (**A**) mark where the intestinal portals are situated. At HH8 cells from region A were located in the intermediate mesoderm and head mesenchyme, whilst cells from region B were located in the intestinal portals and in the heart forming region. (**B** ***i*** **–B** ***iv***) Sections of embryo in (**B**) demonstrated that cells from region A (red) were located in the cranial paraxial mesoderm (CPM) and cells from region B (green) were located in the splanchnic mesoderm (SpM). (**B** ***i*** **–B** ***iii***) Colour channels of sections have been separated into DiI/red on the left hand side panels and DiO/green on the right hand side panels for clearer observation of cell location. (**C**) Labelled cells from region B were also observed in the dorsomedial region of the splanchnic mesoderm, marked with an oval (n = 10). (**D**) DiI labelled descendants of cells from a region of the primitive streak just caudal to the limits of region B were located in the extraembryonic mesoderm (red cells), (n = 21). Scale bars = 50 µm.

After dye labelling of cells in regions A and B, the location of their descendants was analysed using time-lapse imaging to observe the early migration trajectory of these cells during development (Figure 1A–F) and at stage HH8, before the formation of the linear heart tube (Figure 2A–C). In embryos injected at HH3 through to HH3^+^, vital fluorescent dye labelled cells from region A and region B were found to have migrated away bilaterally from the primitive streak by 3 hours post injection (Figure 1C) and demonstrated an anterior-lateral migration pattern. Cells from region B followed a parallel direction to the migration trajectory of cells from region A (Figure 1E and F). By HH8 the labelled descendants of cells from region A (red cells) were located throughout the cranial paraxial mesoderm (CPM) (Figure 2A–Bi*v*). In contrast, the labelled descendants of cells from region B (green cells) demonstrated a migration pattern stereotypical to that previously characterised for CPCs. They were within the bilateral heart fields and by HH8 were located towards the midline at the anterior intestinal portal within the heart forming regions (Figure 2A–B) and the splanchnic mesoderm (Figure 2B *i*–C). At HH8 CPCs, which will contribute to the inflow and outflow myocardium are known to be located within the splanchnic mesoderm [26]. In addition, CPCs from part of the AHF have been described to reside in the dorsomedial region of the splanchnic mesoderm [19]. We observed that some labelled descendants of region B cells (green cells) were located in the dorsomedial region of the splanchnic mesoderm at HH8 (Figure 2C). In order to define the limits of region B we also labelled cells just caudal to region B and observed that labelled descendants of cells from this region (red cells) were located in the extraembryonic mesoderm by stage HH8 (Figure 2D). This suggested that we identified the limits of region B that contribute to progenitors within the splanchnic mesoderm.

These experiments demonstrated that cells from regions A and B (HH3 to HH3^+^) migrate away from the primitive streak with a similar spatiotemporal pattern, however, by stage HH8 were located in distinct parts of the developing embryo. Cells from region A were located throughout the CPM, which has been shown to contribute to the cardiac outflow tract [19], [25]. In contrast, cells from region B demonstrated the same migration pattern to that previously described for CPCs [10] and by stage HH8 were located in the splanchnic mesoderm where CPCs of the PHF and SHF/AHF have been identified.

### Cells which Ingress from Region B of the Primitive Streak are CPCs

Our fate-mapping experiments demonstrated that labelling within our defined region B in stage HH3 though to HH3^+^ embryos resulted in the presence of labelled cells within heart forming regions and the splanchnic mesoderm at stage HH8. To determine whether these cells were CPCs we performed immunohistochemistry to detect expression of Islet-1 (Isl1). Isl1 is a transcription factor, which marks undifferentiated CPCs and is downregulated as cells differentiate and are added to the developing heart [32], [33]. In chicken embryos, Isl1 is expressed throughout the splanchnic mesoderm, including the dorsomedial aspect, and the pharyngeal endoderm at stage HH8. By stage HH12, Isl1 expression is observed in the splanchnic mesoderm underneath the ventral pharynx, and the pharyngeal endoderm but is not observed in cardiac myocardium [25]. Thus in the chicken, Isl1 expression is lost from the differentiating myocardium but remains in the specified but undifferentiated splanchnic mesoderm [34], [25].

**Figure 3:**
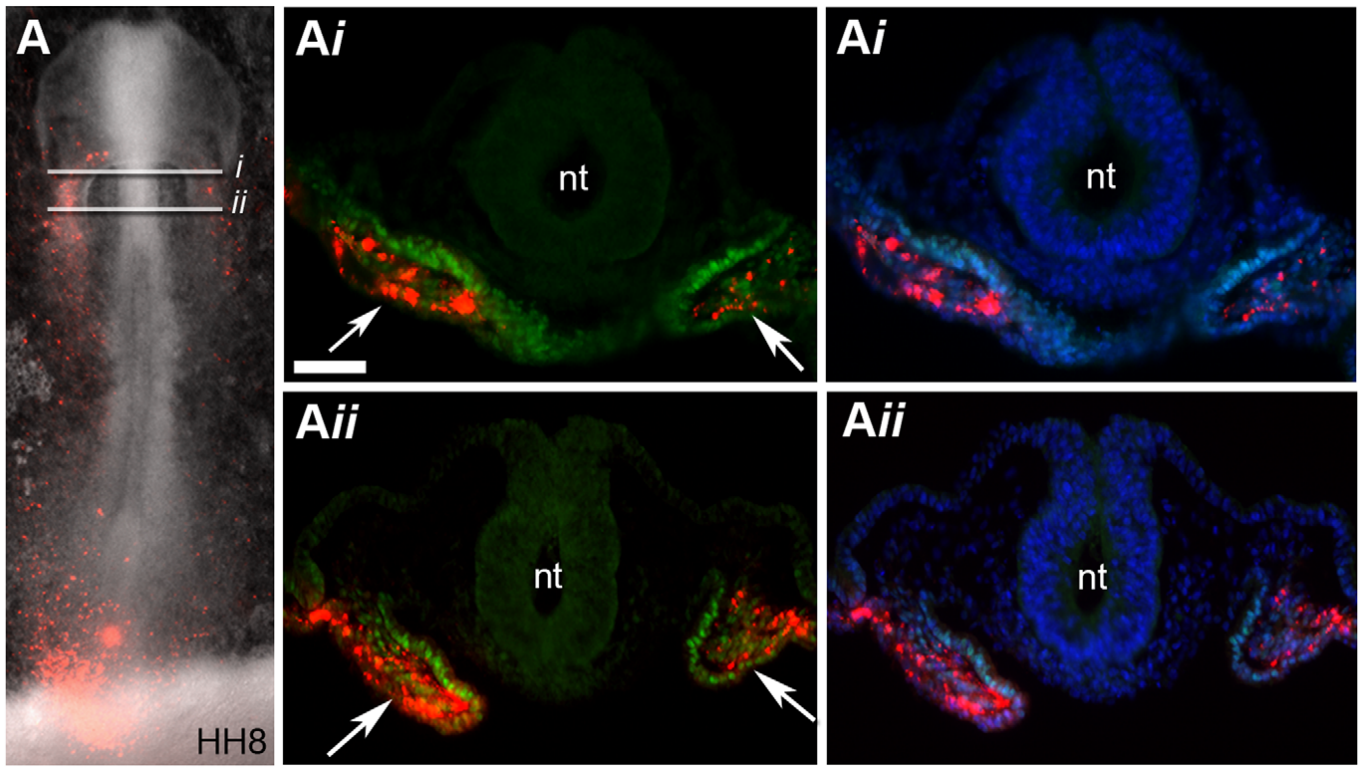
Descendants of cells from region B are located within the splanchnic mesoderm and express Isl1. (**A**) Bright field and fluorescent image showing the location of fluorescently labelled descendants of cells from region B at stage HH8 (red cells). (**A** ***i*** **–A** ***ii***) Sections through embryo in (**A**) were stained for Isl1 protein expression (green). DAPI signal (blue) has been superimposed to provide a clearer picture of the morphology of the tissue. White arrows in (**A** ***i*** **–A** ***ii***) point to the splanchnic mesoderm where fluorescent cells (DiI intercalated within the cell membrane) that express Isl1 are located. Neural tube (nt), (n = 11). Scale bar = 50 µm.

Immunohistochemistry for Isl1 protein on sections from embryos at stage HH8 showed that vital fluorescent dye labelled descendants of cells from region B were located within the splanchnic mesoderm at stage HH8, including the dorsomedial region (Figure 2C), and expressed Isl1 protein (Figure 3A–A *ii*). This confirmed that region B derived cells give rise to Isl1 positive cells (CPCs) located within the splanchnic mesoderm and that region B at stage HH3 though to HH3^+^ is the ingression site of these cells.

### Fate Mapping of Region A Cells to Linear Heart Tube Stages of Development

**Figure 4:**
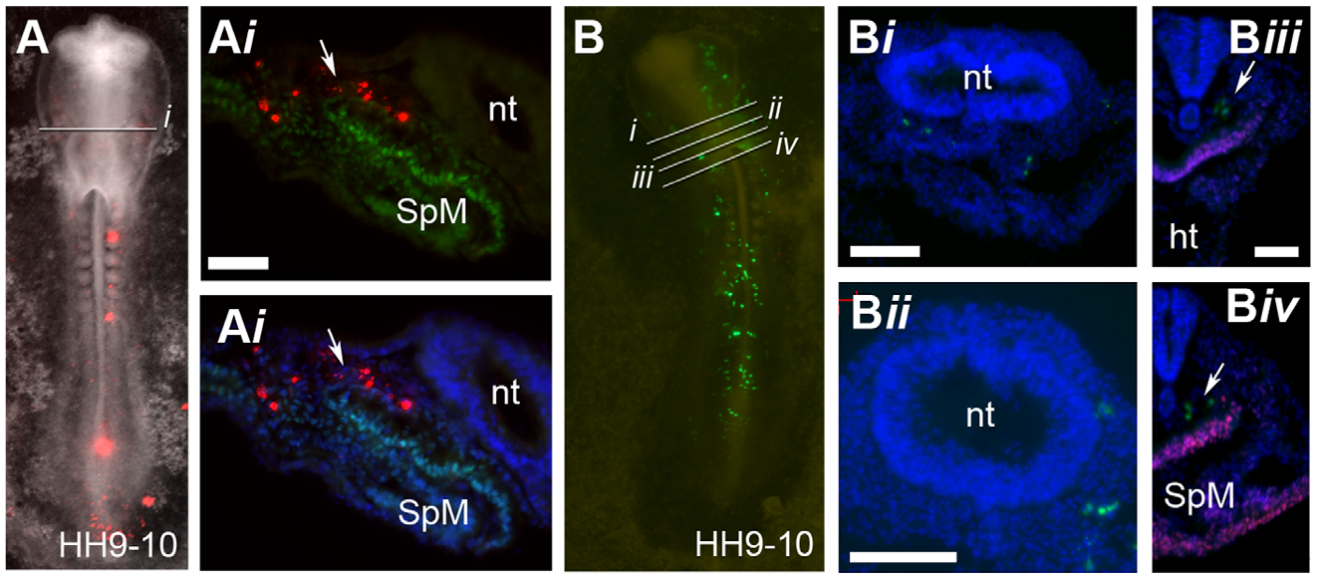
Descendants of cells from region A are located within the CPM at stage HH9-10. (**A**) Bright field and fluorescent image showing the location of fluorescently labelled descendants of cells from region A at stage HH9-10 (red cells). (**A** ***i***) Section through embryo in (**A**) showing location of labelled cells and Isl1 protein expression (green). DAPI signal (blue) has been superimposed to provide a clearer picture of the morphology of the tissue. White arrows in (**A** ***i***) point to the CPM where fluorescent cells are located. (**B**) Bright field and fluorescent image of embryo at stage HH9-10 showing location of CAG-GFP labelled cells (green cells) which were grafted within region A of the primitive streak at stage HH3 though to HH3^+^. (**B** ***i*** **–** ***iv***) Section of grafted embryo (**B**)**.** (**B** ***iii*** **–** ***iv***) Sections stained for Isl1 protein expression (red). Arrows indicate the location of CAG-GFP cells in the CPM. At stage HH9-10 CAG-GFP labelled cells grafted to region A were located within the CPM (n = 5). Splanchnic mesoderm (SpM), neural tube (nt), heart (ht). Scale bars (**A** ***i***)**,** and (**B** ***iii*** **–** ***iv***) = 50 µm, (**B** ***i*** **–** ***ii***) = 100 µm.

In stage HH8 embryos, fluorescent dye labelled descendants of cells from region A were located within the CPM (Figure 2A–B *iv*). To determine the location these cells once the linear heart tube is present we analysed the location of these cells in embryos at stage HH9-10. At this stage descendants from region A were located in the CPM (Figure 4A–*A* *i*). To confirm the dye-labelling results we used an alternative approach and generated transplantation chimeras containing grafts derived from transgenic CAG-GFP chicken embryos. Grafts of primitive streak cells carrying a GFP transgene [29]were dissected from region A of donor embryos and transferred to region A of wild-type, stage-matched host embryos (HH3 though to HH3^+^). Grafting of GFP expressing region A cells of the primitive streak into host chicken embryos produced results similar to those obtained with vital fluorescent dye labelling. Cells grafted to region A were located within the CPM by HH10 (Figure 4B *i*–B*iv*). Thus, both cell tracing techniques demonstrated that cells within the CPM, originated from region A of the primitive streak at stage HH3 though to HH3.

**Figure 5:**
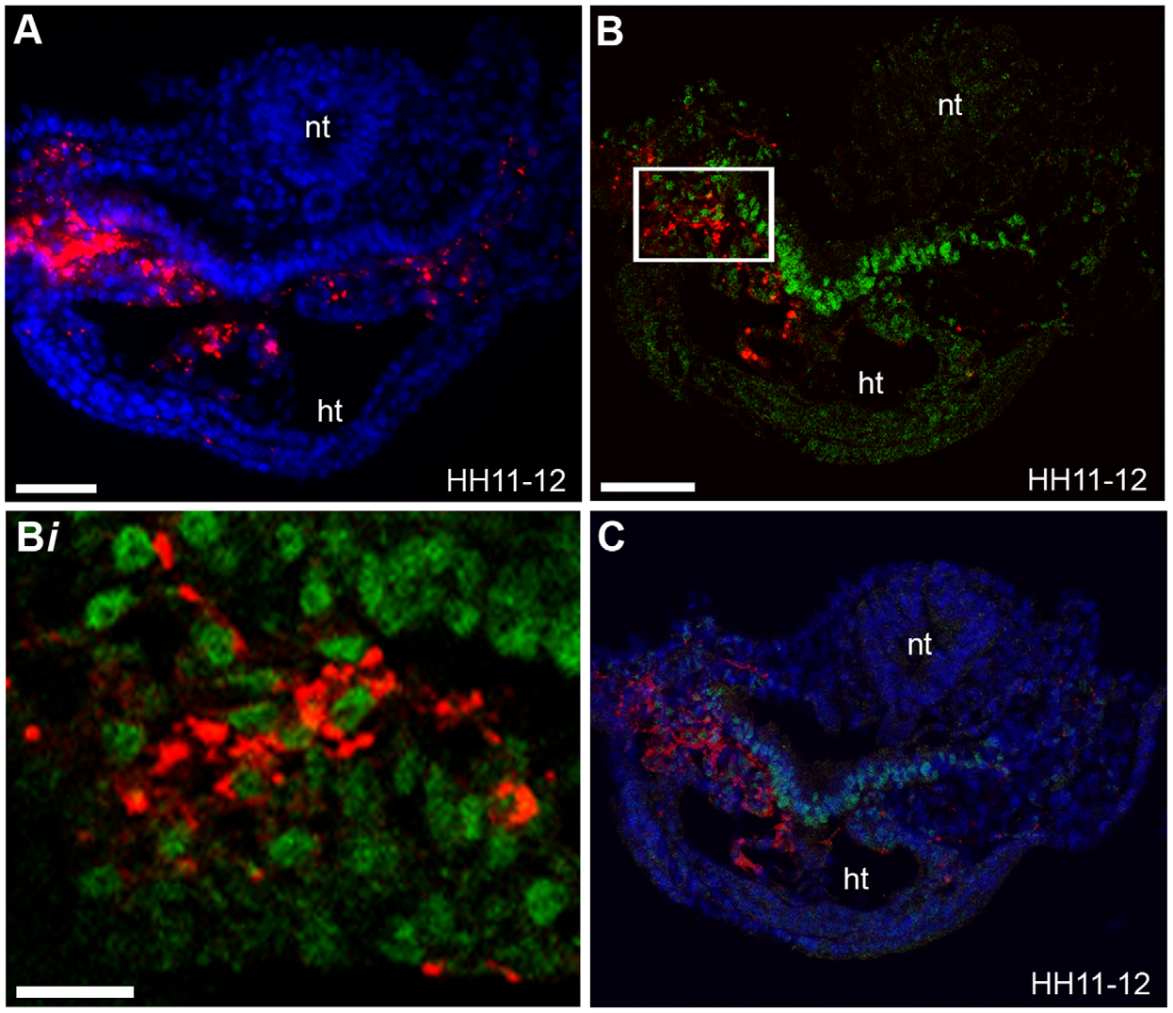
Descendants of cells from region B are located within the splanchnic mesoderm and heart. (**A–B** ***i***) Sections from labelled stage HH11-12 embryos. (**A–B**) Sections at the level of the caudal pharynx showed that fluorescent descendants of cells from region B (red cells) were located in the splanchnic mesoderm and within the heart (ht). (**B–B** ***i***) Isl1 protein expression is indicated (green). Images were taken on a confocal microscope, **(C)** DAPI signal (blue) has been superimposed to provide a clearer picture of the morphology of the tissue. (**B** ***i***) Higher magnification of the boxed region in (**B**). Labelled cells located in the splanchnic mesoderm with DiI intercalated within the cell membrane express Isl1. Neural tube (nt), (n = 20). Scale bars (**A**) = 50 µm, (**B**) = 60 µm, (**B** ***i***) = 13 µm.

**Figure 6:**
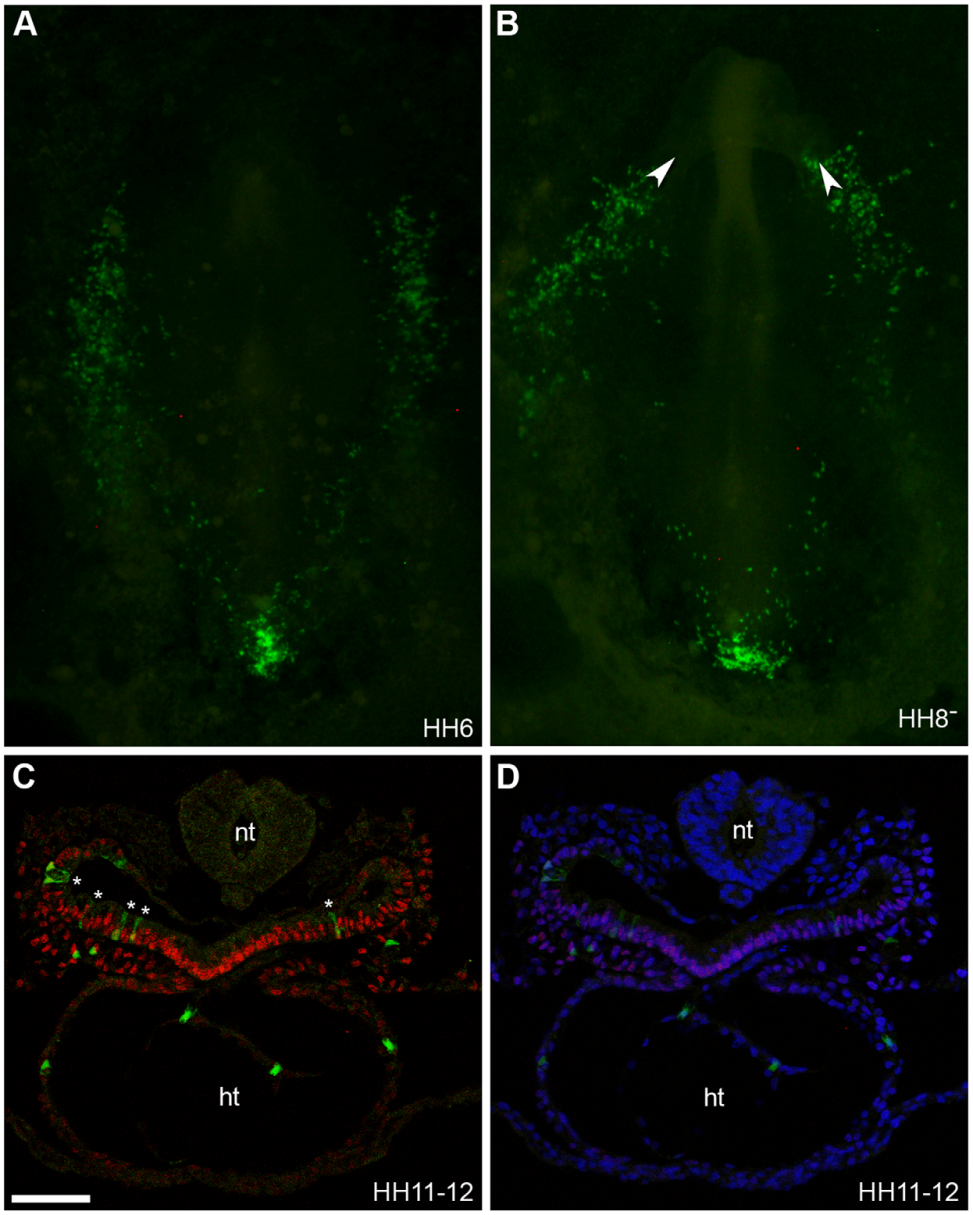
CAG-GFP cells grafted within region B are located in the splanchnic mesoderm and heart. (**A–B**) Bright field and fluorescent images of embryos at stage HH6 (**A**) and HH8^-^ **(B)** showing location of CAG-GFP labelled cells (green cells) which were grafted within region B of the primitive streak at stage HH3 though to HH3^+^. White arrow heads in (**B**) mark the location of the intestinal portals. (**C**) Section of grafted embryo stained for Isl1 protein expression (red). (**D**) DAPI signal (blue) has been superimposed to provide a clearer picture of the morphology of the tissue. At stage HH11-12 CAG-GFP labelled cells grafted to region B were located within the pharyngeal endoderm (cells marked with white asterix), splanchnic mesoderm and the heart (ht). Images were taken on confocal microscope. Neural tube (nt), (n = 19). Scale bar = 60 µm.

**Figure 7:**
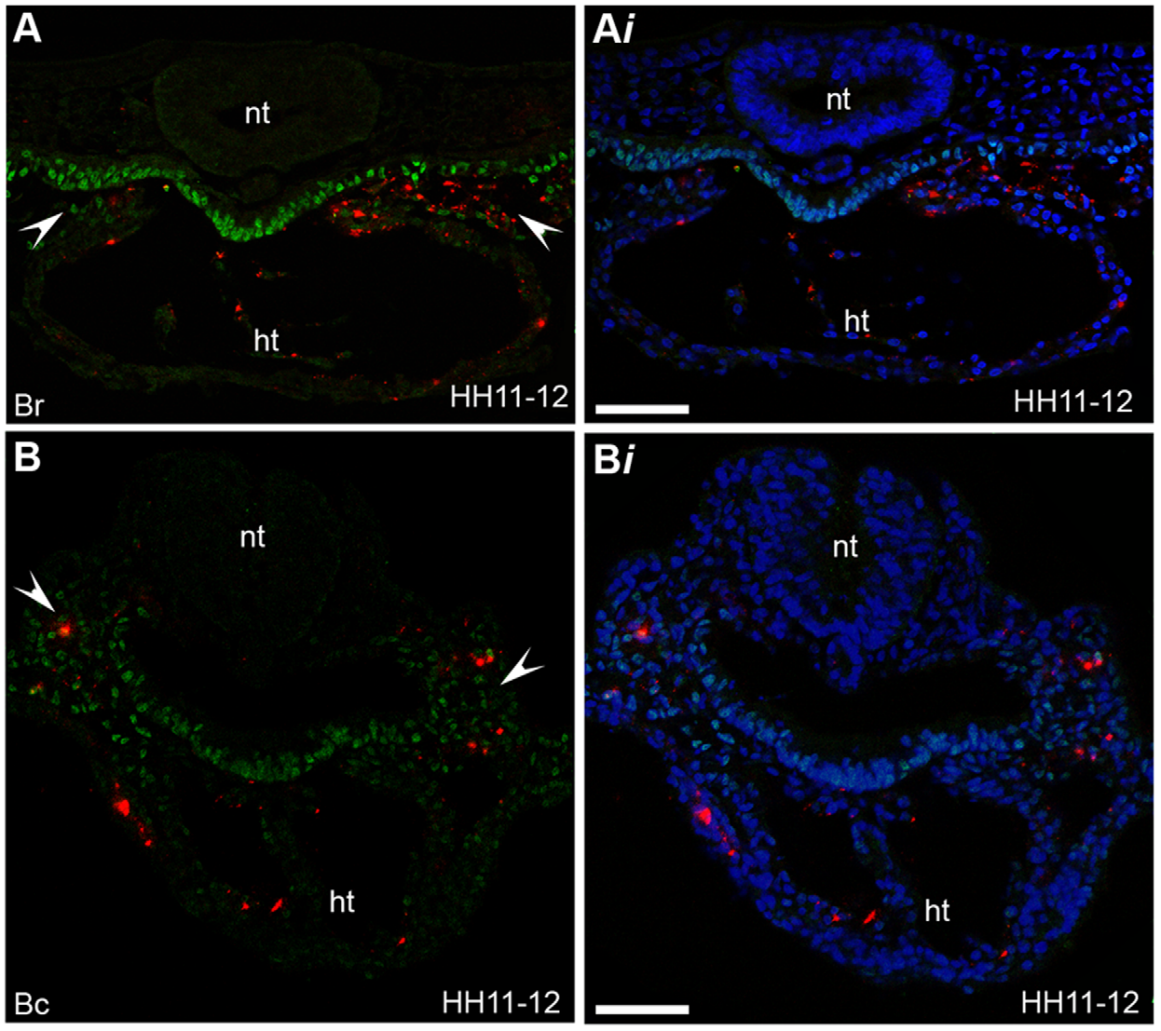
Region B is the ingression site of CPCs located within the splanchnic mesoderm and heart. (**A–B** ***i***) Sections of labelled embryos at stage HH11-12 stained for Isl1 protein expression (green). (**A**) Fluorescent descendants of cells (red cells) labelled in rostral subdivisions within region B (Br) are located within the splanchnic mesoderm (indicated with white arrow heads) and the heart (ht). (**B**) Fluorescent descendants of cells (red cells) labelled in caudal subdivisions within region B (Bc) are located within the splanchnic mesoderm (indicated with white arrow heads) and the heart (ht). (**Ai, Bi**) DAPI signal (blue) has been superimposed to provide a clearer picture of the morphology of the tissue. Images taken on confocal microscope, neural tube (nt) (n = 31). Scale bars = 60 µm.

**Figure 8:**
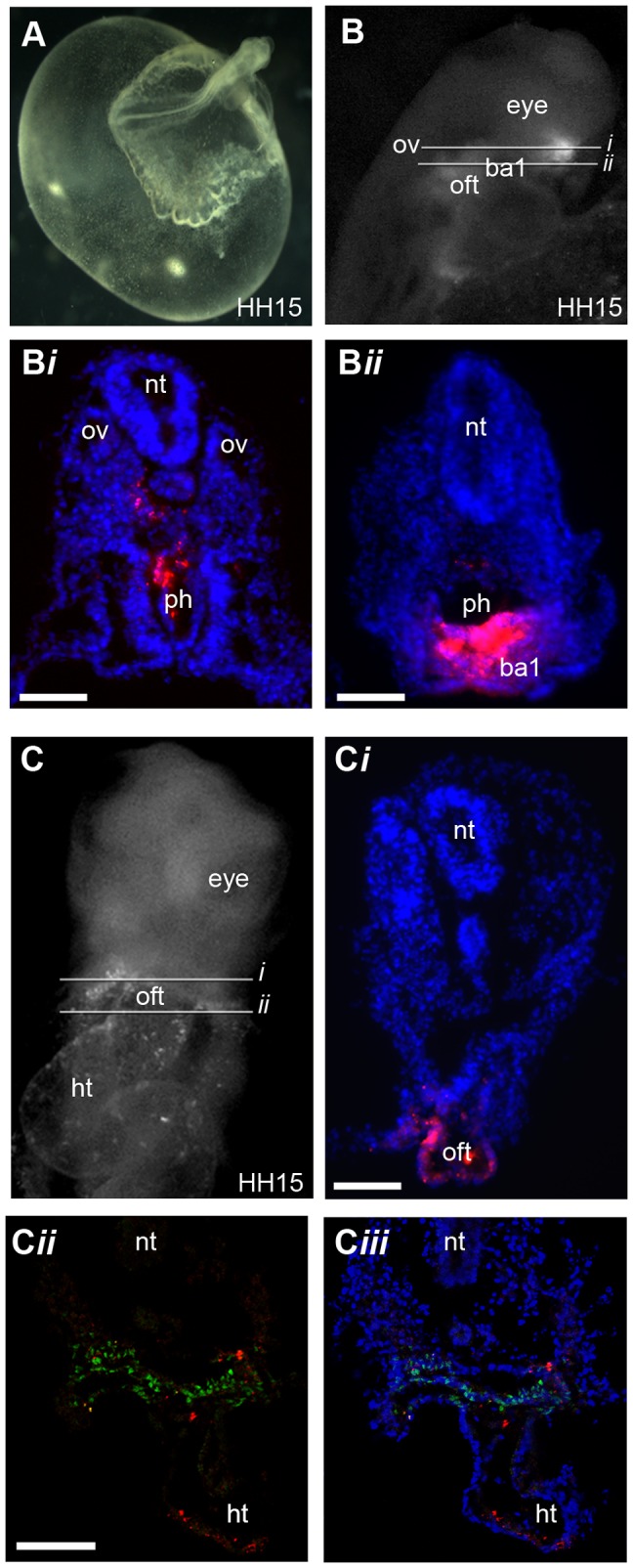
Fate mapping of fluorescent descendants of cells from region A and B to stage HH15. (**A**) Bright field image of an embryo grown in MC culture**.** (**B**) Black and white fluorescent image showing the location of fluorescently labelled descendants of cells from region A. (**B** ***i*** **–B** ***ii***) Sections of (**B**) demonstrate that cells from region A are located in the first branchial arch (ba1), (n = 5). (**C**) Black and white fluorescent image showing the location of fluorescently labelled descendants of cells from region B (**C** ***i*** **–C** ***ii***) Sections of (**C**) show that cells from region B are locate in the splanchnic mesoderm and the outflow tract (oft), (n = 6). (**C** ***ii***) Isl1 protein expression is shown in green and (**C** ***iii***) DAPI signal (blue) has been superimposed to provide a clearer picture of the morphology of the tissue. (**C–C** ***iii***) Images were taken on confocal microscope. Neural tube (nt), heart (ht), otic vesicle (oc), pharynx (ph).Scale bars (**B** ***i***–**B** ***ii***, **C** ***i***) = 50 µm, (**C** ***ii***) = 60 µm.

**Figure 9:**
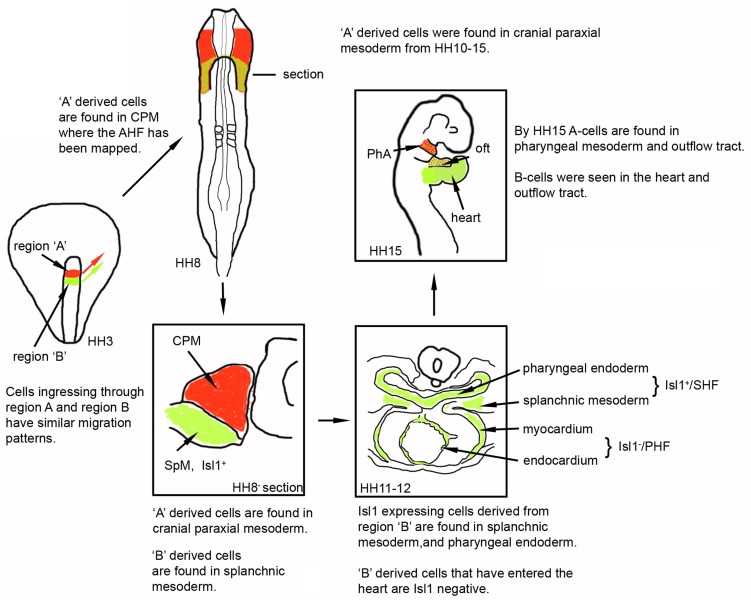
Model illustrating the fate of cells derived from regions A and B. The location of regions A (red) and B (green) within the anterior primitive streak of HH3 and HH3^+^embryos and their migration patterns are indicated by red and green arrows. At HH8 ‘A’ derived cells are located in the CPM and ‘B’ derived cells are found in splanchnic mesoderm. This is schematically shown in a section. By HH11-12 region ‘B’ descendants have segregated into Isl1 positive cells, which are found in the pharyngeal endoderm and splanchnic mesoderm, and an Isl1 negative lineage found in the linear heart tube. Region ‘A’ descendants continue to be found in CPM (not indicated). By HH15 the region ‘B’ derived cells are found in the outflow tract, heart and splanchnic mesoderm, whilst region ‘A’ descendants are seen in the cranial paraxial mesoderm, pharyngeal arches and outflow tract.

### Region B Cells Contribute to Both the Linear Heart Tube and a Population of CPCs within the Splanchnic Mesoderm

We next wanted to determine whether region B derived CPCs contributed only to the PHF or also to the SHF/AHF within the splanchnic mesoderm. It has been previously demonstrated that following linear heart tube stages, Isl1 expressing cells within the splanchnic mesoderm contribute to the distal part of the first pharyngeal arch as well as to the outflow myocardium [25]. Furthermore, splanchnic mesoderm explants from stage HH10 embryos express Isl1 in vitro, thus can undergo further cardiac differentiation [19]. We therefore analysed the location of fluorescent region B descendants in embryos at stage HH11-12, after formation of the primary heart tube. By this stage CPCs of the PHF have differentiated, lost Isl1 expression and are located in the linear heart tube. Sections from stage HH11-12 embryos at the level of the caudal pharynx demonstrated that there were some vital fluorescent dye labelled cells located in the splanchnic mesoderm, which expressed Isl1 and others, not expressing Isl1, which were located within the - linear heart tube (Figure 5A–B *i*). The fluorescent cell descendants located in the linear heart are PHF derived cells, which have differentiated and entered the heart. The fluorescent Isl1-positive descendants, which were still located in the splanchnic mesoderm, are CPCs which will be added to the developing heart later and are progenitors from what has been described to be the SHF [17] and part of the AHF [25].

To confirm the dye-labelling results grafts of primitive streak cells carrying a GFP transgene [29] were dissected from region B of donor embryos and transferred to region B of wild-type, stage-matched host embryos (HH3 though to HH3^+^). Grafting of GFP expressing region B cells of the primitive streak into host chicken embryos produced results similar to those obtained with vital fluorescent dye labelling. Cells grafted to region B migrated from the primitive streak in an anterior-lateral lateral pattern and were located within the bilateral heart fields by HH5- 6 (Figure 6A). At HH7-8 GPF cells were located within the future heart forming regions (Figure 6B) and by stage HH11-12 we observed that CPCs were located within the primary heart tube, the pharyngeal endoderm and the splanchnic mesoderm (Figure 6C, D). Thus, both cell tracing techniques demonstrated that CPCs of the linear heart tube (PHF) and CPCs, which remain within the splanchnic mesoderm and will be added to the heart later during development, originated from region B of the primitive streak at stage HH3 though to HH3^+^. Using this approach we observed some fluorescent cells within the pharyngeal endoderm. We did not observe this when performing fluorescent dye labelling/injections, suggesting that there are only very few prospective endoderm cells present in region B at HH3 to HH3^+^. This is consistent with previous work, which mapped primitive streak cells from HH3^+^ to HH7-9 and showed that most prospective endoderm cells reside in the anterior streak at stage HH4^–^ and HH4 [35].

To determine whether CPCs, which are added to the developing heart at different times, ingressed from the same location within region B we injected vital fluorescent dye into subdivisions of region B (Table 1). By doing this, we examined the fate of cells from subdomains within the rostral part of region B, designated as region Br, or the caudal part of region B, designated as Bc. Analysis of embryos at stage HH11-12 showed that fluorescent cell descendants of cells from all subdivisions within region Br and Bc were located in both the splanchnic mesoderm and in the linear heart (Figure 7A–B *i*). These results demonstrated that region B of the primitive streak was the site of ingression at HH3 though to HH3^+^ of CPCs which contribute to the primary heart tube and of Isl1-positive CPCs which remained undifferentiated and were located where the SHF and part of the AHF has been previously described [17], [25]. Consistent with previous work [18], [17], [25] the undifferentiated CPCs, which remained within the splanchnic mesoderm at HH12 are likely to contribute to the outflow myocardium at later stages.

### Region A Cells Contribute to the First Branchial Arch and Region B Cells Contribute to the Outflow Tract in Stage HH15 Embryos

To confirm that the progenitor cells located within the CPM and the splanchnic mesoderm by stage HH10-12 have the potential to contribute to the outflow myocardium we maintained fluorescent dye labelled embryos in a MC culture system [28], which allowed analysis of the location of fluorescent labelled descendants of cells from region A and B past stage HH12 (Figure 8A). By allowing embryos to develop to stage HH15 we observed that fluorescent labelled descendants of cells from region A, which were located in the CPM at stage HH10, were located in the CPM and the first branchial arch (BA1) at stage HH15 (Figure 8B *i–*B*ii*). Previous fate mapping experiments have mapped AHF progenitors to the CPM in stage HH8 chicken embryos and have demonstrated that these progenitors contribute to the first branchial arch as well as the cardiac outflow tract [19], [25]. Thus our results are consistent with previous fate mapping studies and demonstrate that region A of the primitive streak at stage HH3 (PS2) and HH3^+^ (PS4) is the ingression site of progenitor cells from the AHF.

In addition, we observed that fluorescent labelled descendants of cells from region B were located in the splanchnic mesoderm, outflow tract and linear heart tube at stage HH15 (Figure 8C–C *ii*). This result recapitulates previous fate mapping studies performed in embryos from stage HH8 onwards, which demonstrate that cardiac progenitors located within the splanchnic mesoderm at stage HH8-12 contribute to the outflow tract [17], [26], [25]. These progenitors have been referred to as being from the SHF [17], [26] and part of the AHF [25]. Thus, our results demonstrate that CPCs, which contribute to what is described to be the PHF, the SHF and part of the AHF, originate from region B of the primitive streak at stage HH3 (PS2) through to HH3^+^ (PS4) This identifies a common origin of bilateral heart field progenitors, which contribute to the linear heart tube and subsequently to the inflow and outflow myocardium.

## Discussion

In the present study, we used chicken embryos to perform fate-mapping experiments of CPCs, which contribute to the outflow myocardium and have determined the origin of ingression of these cells from the HH3 (PS2) to HH3^+^ (PS4) primitive streak. We have defined new regions, region A and region B from the anterior-middle primitive streak that can easily be delineated using an eye piece reticule with a NE1scale. At stage HH 3 through to HH3^+^ the limits of region A and region B change slightly as the primitive streak increases in length (Table 1).

Our results demonstrate that region A is the site of ingression of cells which contribute to the CPM by stage HH8 and to the first branchial arc at stage HH15. Furthermore, they show that region B is the site of ingression of CPCs, which by HH8 are located in the splanchnic mesoderm, including the dorsomedial region, described to be part of the AHF [25]. By HH12 some of the region B derived cells contributed to the linear heart tube and others remained in the splanchnic mesoderm underlying the caudal pharynx, where the SHF has previously been described to be located [17], and by stage HH15 were present in the outflow tract. This study is the first to map the origin of CPCs, which contribute to the outflow myocardium, in early primitive streak stage embryos.

We have delineated region A in the early chicken gastrula, with precisely defined limits, that is the location of ingression of progenitors which contribute to the CPM by stage HH8 and are part of the AHF. This is in agreement with previous fate maps which showed that the anterior end of the primitive streak in HH3 to HH4 embryos contributes to prospective head mesenchyme [2], [35]. In addition, fate mapping studies have revealed that some cells within the CPM form part of the AHF and contribute to the myocardium and endocardium of the cardiac outflow tract [19], whilst others generate head skeletal muscles [20] (Figure 9).

Furthermore, our study demonstrated that CPCs, which will generate what has been identified as PHF, SHF and part of the AHF, migrated out simultaneously from region B of the early primitive streak during gastrulation. It is possible that these CPCs are a mixed population of pre-specified and discrete progenitor cells; however, in the absence of specific molecular markers there is currently no evidence to support this notion. Alternatively, region B derived CPCs may constitute a population of cells, which have the potential to contribute to the PHF, the SHF and parts of the AHF within the cardiogenic mesoderm. In this latter scenario we propose that there are mechanisms, which pattern the cardiogenic mesoderm resulting in spatio-temporal segregation. This would then lead to the differentiation of some CPCs and the formation of the linear heart tube (CPCs from the PHF), while other CPCs remain undifferentiated within the splanchnic mesoderm and constitute a secondary lineage of CPCs. These will be added to the linear heart tube later during development to contribute to the outflow myocardium (Figure 9). The lack of genetic and lineage-specific markers for these early progenitors in the cardiogenic mesoderm could explain why in chicken embryos the precise boundaries and molecular identities of the different heart fields have not been completely clear before stage HH12.

It has been proposed that in stage HH8 chicken embryos the cardiogenic mesoderm is organised in cranio-caudal stripes with the inflow precursors located in the lateral part while the outflow precursors are located in the medial part of cardiogenic mesoderm [26]. The CPCs, which contribute to the inflow and outflow myocardium, are thus contiguous within the cardiogenic mesoderm and thus mechanism/s which pattern the cardiogenic mesoderm may already be active at stage HH8. Furthermore, it has been suggested that the inflow/outflow organisation of the heart is established in an anterior-posterior manner by mechanisms that include retinoic acid signalling [36]. Retinoic acid signalling is believed to divide the cardiac field between stages HH7-8 into domains containing precursors, which contribute to the inflow or outflow tracts of the heart [37]. Our results support the existence of mechanism which pattern the cardiac field and also demonstrate that a mixed population of CPCs, either discrete or with multiple potentials, exists by stage HH3 and ingresses through region B, emerging from the primitive streak with an anterior-lateral migration pattern. In addition, our data supports the idea that there is a bilateral heart field within the splanchnic mesoderm that is spatio-temporally defined by complex patterning [23], [26]. Moreover, a retrospective clonal analysis in the mouse suggested that the first (PHF) and second (AHF) lineages of cardiac progenitors originate from a common precursor population that segregates prior to the cardiac-crescent stage, just before the fusion of the cardiogenic mesoderm [38]. The detailed mechanisms regulating patterning and cell lineage segregation are under investigation [37], [39] and they are likely to include inductive signals and extrinsic cues from the surrounding tissue as those that regulate head muscle patterning [40], [41]. In addition, inductive signals which control the migration [9], [10] or differentiation of CPCs [42], [43], [44], or which are known to be important for the maintenance of the SHF/AHF once established [33], [45], [46] may also contribute to the segregation of different lineages.

A limitation of using embryos in culture is the difficulty in visualising labelled cells and performing fate-mapping experiments in late stage embryos. However, our aim was to examine the origin of outflow myocardium at early primitive streak stages. Unfortunately, early stages of avian development are not well suited for *in ovo* studies. However, by fate-mapping cells in cultured embryos, we have been able to accurately determine the placement and movement of labelled cells up to stage HH15 when branchial arches and outflow tract structures are already present. Furthermore, the two complementary approaches we used to label cells (vital fluorescent dye injection and transplants of GFP fluorescent grafts) gave similar results and are in agreement with results obtained from fate maps of outflow myocardium progenitors previously performed in chicken embryos from stage HH8 onwards [18], [25], [26]. Therefore our results suggest that CPM cells which contribute to the AHF and the outflow myocardium ingress within region A of the primitive streak at stage HH3 through to HH3^+^, and CPCs of the PHF, SHF and part of AHF ingress within region B at stage HH3 through to HH3^+^. This study maps for the first time the origin of cells that contribute to the outflow tract myocardium from the early primitive streak, thus contributing to a better understanding of the origin and behaviour of CPCs. It will be interesting to elucidate the molecular mechanism/s responsible for the complex patterning of the cardiac mesoderm and the spatio-temporal segregation of CPC lineages.
